# Expected imaging findings and postoperative complications of median sternotomy

**DOI:** 10.1590/0100-3984.2024.0094

**Published:** 2025-02-11

**Authors:** Camila Pietroski Reifegerste, Bruno Lima Moreira, Renan Aguena Arakaki, Augusto Kreling Medeiros

**Affiliations:** 1 BP Medicina Diagnóstica, Hospital Beneficência Portuguesa de São Paulo, São Paulo, SP, Brazil.

**Keywords:** Sternotomy, Postoperative complications, Diagnostic imaging, Mediastinitis, Surgical wound infection, Esternotomia, Complicações pós-operatórias, Diagnóstico por imagem, Mediastinite, Infecção da ferida cirúrgica

## Abstract

A typical approach for many cardiovascular surgical procedures is median sternotomy. Despite advances in surgical techniques and postoperative care, complications still occur in 0.5–5.0% of cases. Radiological assessment of the chest after sternotomy is a challenge, because normal findings resulting from surgical trauma can resemble complications. This pictorial essay aims to provide an overview of normal postoperative imaging findings and the spectrum of complications that can arise, including surgical material-related issues, hematomas, bone complications, and infections. Diagnosis and management require careful interpretation of imaging studies, considering the recovery timeline and clinical status of the patient. With the increasing number of cardiothoracic surgical procedures, this is essential knowledge for radiologists, allowing them to contribute to an accurate diagnosis and the appropriate management of patients.

## Abstract (pt)

Uma abordagem típica para muitas cirurgias cardiovasculares é a esternotomia mediana. Mesmo com os avanços nas técnicas cirúrgicas e nos cuidados pós-operatórios, complicações ainda ocorrem em 0,5–5% dos casos. A avaliação radiológica do tórax após esternotomia é um desafio, pois os achados normais decorrentes do trauma cirúrgico podem se assemelhar a complicações. Este ensaio iconográfico tem como objetivo fornecer uma visão geral dos achados normais de imagem no pós-operatório e do espectro de complicações que podem surgir, incluindo complicações relacionadas a materiais cirúrgicos, hematomas, complicações ósseas e infecções. O diagnóstico e o manejo requerem uma interpretação cuidadosa dos estudos de imagem, considerando o tempo de recuperação do paciente e seu estado clínico. Com o aumento do número de procedimentos cirúrgicos cardiotorácicos, este é um conhecimento essencial aos radiologistas, para que possam contribuir para um diagnóstico adequado e o manejo correto dos pacientes.

## INTRODUCTION

Median sternotomy is the standard surgical approach for various cardiovascular conditions. Despite advances in surgical techniques and postoperative care, complications occur in 0.5–5.0% of cases. Such complications fall into four main categories: surgical material-related complications; hematoma; bone complications; and infections. Because clinical symptoms are frequently nonspecific, imaging is crucial for the postoperative evaluation and detection of these complications^(1,2)^. This pictorial essay aims to depict the normal imaging appearance and the main complications following median sternotomy.

## STERNOTOMY CLOSURE TECHNIQUES

**Figure 1:**
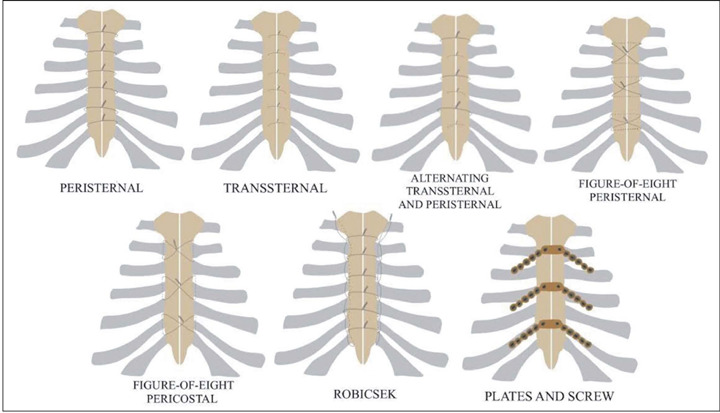
Median sternotomy closure techniques.

**Figure 2:**
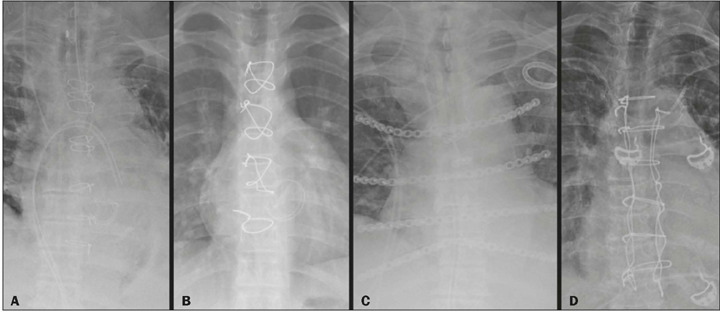
Sternum closure techniques (chest X-ray images). **A:** Transsternal suture. **B:** Figure-of-eight configuration. **C:** Plates and screws. **D:** Robicsek closure.

For sternotomy closure, two configurations—the trans-sternal—peristernal; and the single transsternal—are commonly used for their stiffness, ease of revision, and cost-effectiveness. However, they carry risks like wire fracture and pose challenges in cases involving osteoporotic bone. For revision sternotomy or in patients with osteoporosis, the Robicsek procedure may be applied, providing stability by reinforcing lateral portions of the sternum. The figure-of-eight configuration is another option for sternal closure. In patients at high risk for mediastinitis, plates and screws offer more rigid fixation^(1,2,3)^, as illustrated in Figures 1 and 2.

## EXPECTED POSTOPERATIVE FINDINGS

**Figure 3:**
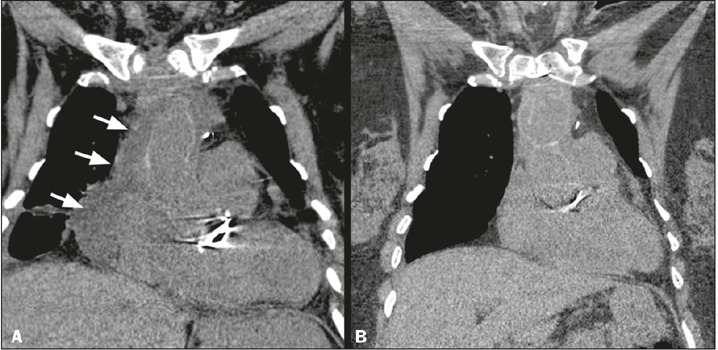
Normal postoperative sternotomy findings (coronal CT images; mediastinal window). **A:** Immediate postoperative period. Mediastinal fluid collection is observed surrounding the right atrium and thoracic aorta (arrows), which may be an expected finding in a normal recent postoperative period (up to three weeks). **B:** Follow-up CT performed three months later. Complete resorption of the collection is noted.

Interpreting imaging studies in the first 2–3 weeks after sternotomy is challenging because normal healing findings can resemble complications like mediastinitis. Such findings include the following (Figure 3): edema (fat stranding); hypodense (fluid) or hyperdense (blood) accumulations in the presternal or retrosternal compartments; pneumomediastinum; and sternal separation. A pneumomediastinum should be reabsorbed within 1–2 weeks, while edema and fluid or blood accumulations usually resolve within 2–3 weeks. Sternal gaps of up to 4 mm can persist for up to 12 months without indicating sternal non-union, and callus formation can begin in postoperative month 3^(1,4)^.

## FINDINGS OF POSTOPERATIVE COMPLICATIONS

### Surgical material-related complications

**Figure 4:**
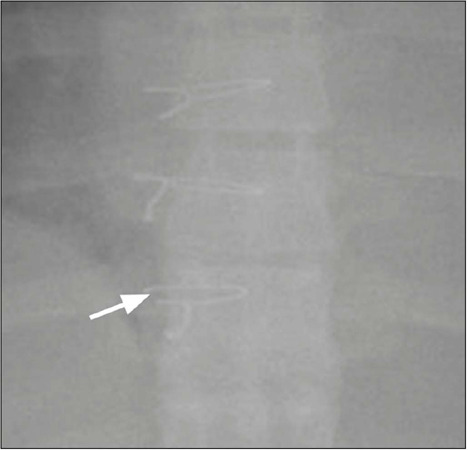
Sternal wire fracture (magnified chest X-ray image): discontinuity on the right side of the last sternal metallic wire (arrow).

**Sternal wire fracture** – Sternal wire fractures have an estimated incidence of 2–3%. They are identified on radiographs as a focal defect (Figure 4). If there is uncertainty, a computed tomography (CT) scan may be performed^(1)^.

**Figure 5:**
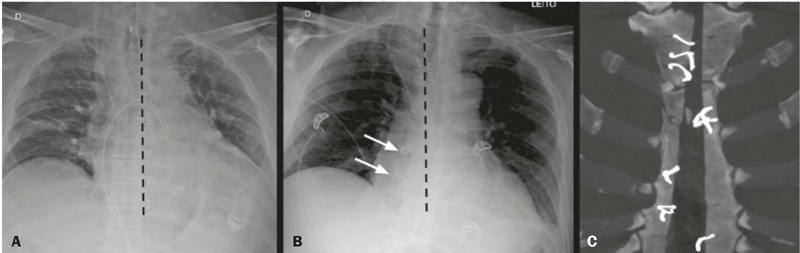
Sternal dehiscence. **A:** Frontal chest X-ray in the immediate postoperative period, showing alignment of the sternal wires. **B:** Frontal chest X-ray on post-operative day 6, showing right lateralization of the two last sternal wires (arrows), suggestive of dehiscence. **C:** Coronal CT image (maximum intensity projection reconstruction; bone window) confirming the dehiscence of the sternum.

**Sternal wire migration and dehiscence** – In sternal wire migration, wires remain intact, with radiographs showing lateral displacement. Dehiscence involves sternal separation, with intact sternal wires migrating along with the displaced sternal fragment, typically within 1–40 days after surgery, with an incidence of 1–3%. This may lead to serious complications, including infection and wire displacement towards the aorta. Risk factors include age > 60 years, osteopenia, postoperative infection, and diabetes. Radiographic findings may precede the clinical diagnosis by 3–6 days, and lateral displacement (of 2 cm on average) of two or more sternal wires is usually observed in cases of sternal dehiscence (Figure 5). In some cases, a midsternal lucent stripe thicker than 3 mm is seen. Because X-rays offer limited bone assessment, CT may be necessary^(1,2,5)^.

**Figure 6:**
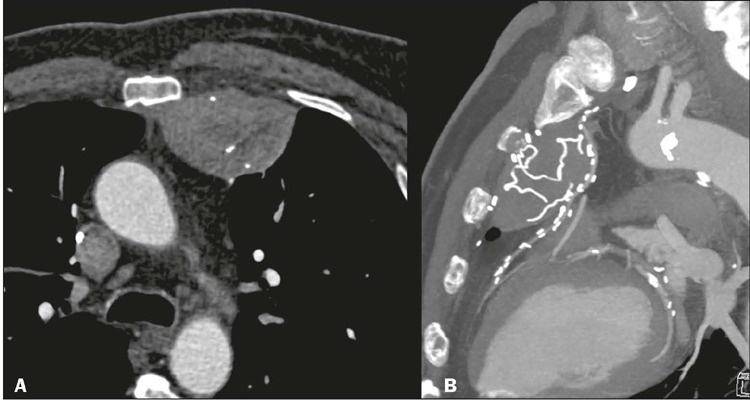
Gossypiboma. Axial CT image; mediastinal window **(A)** and sagittal CT image; maximum intensity projection reconstruction; mediastinal window **(B),** showing heterogeneous oval mass, with regular contours encompassing a hyperdense linear image (radiopaque marker), located in the prevascular mediastinum, consistent with a gossypiboma.

**Gossypiboma** – Intrathoracic gossypiboma refers to a foreign object, like a mass of cotton matrix or a sponge, left within the thoracic cavity during surgery, surrounded by a foreign body reaction. Patients may be asymptomatic or present with chest pain, cough, dyspnea, or hemoptysis. On CT, a gossypiboma typically appears as a homogeneous or, more commonly, heterogeneous, rounded or oval mass with regular contours, with or without peripheral enhancement (Figure 6). Other potential imaging features include a wrinkled appearance, hyperdense linear image (radiopaque marker), spongiform appearance, and areas of high and low attenuation^(6)^.

### Hematoma

**Figure 7:**
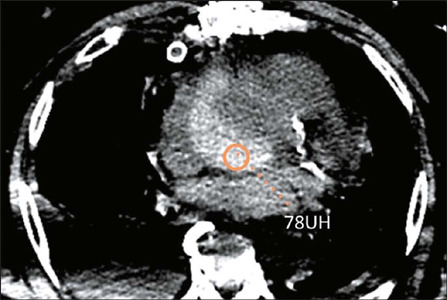
Mediastinal hematoma. Axial CT image (mediastinal window) showing a hyperdense collection (mean attenuation: 78 HU) surrounding the lower third of the ascending aorta, with a maximum thickness of 27 mm, consistent with a mediastinal hematoma.

Hematomas usually occur in the early postoperative period after median sternotomy, developing in the prevascular mediastinum or chest wall. The pathophysiological mechanism is multifactorial. Operations on large vascular structures, traumatic sternal fracture, increased intrathoracic pressure, anticoagulation, and other factors may be implicated. On CT, hematomas usually correspond to well-circumscribed hyperdense collections (Figure 7). Surgical re-exploration may be required in cases of significant bleeding, rapid expansion, or a significant mass effect^(1,2)^.

### Bone complications

**Figure 8:**
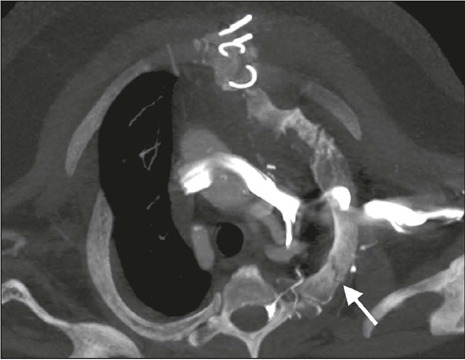
Rib fracture (axial oblique CT image; maximum intensity projection reconstruction; bone window). Trace of complete fracture in the posterior aspect of the left first rib (arrow) after sternotomy.

**Fractures** – Bone fractures occur in up to 13% of patients, usually involving the posterior portion of the 1st or 2nd ribs (Figure 8), and are associated with excessive intraoperative retraction and obesity. Cartilaginous fractures can also occur^(1)^.

**Figure 9:**
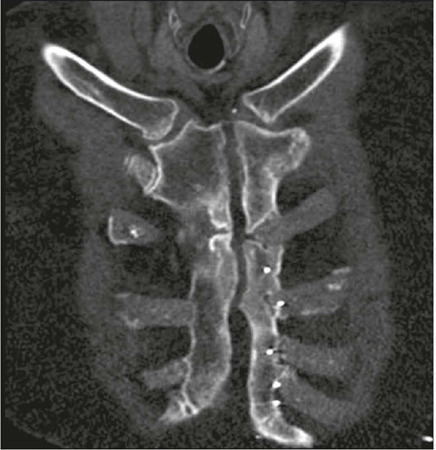
Sternal non-union (coronal CT image; bone window). Separation of sternal fragments, with corticated margins, visualized on a CT scan performed 18 months after sternotomy.

**Sternal non-union** – Sternal non-union (with an incidence of 0.5–3.0%) is defined as the failure of sternal fragments to fuse. Risk factors include osteoporosis, chest irradiation, corticosteroid use, obesity, and internal mammary artery harvest^(1)^. Clinically, it presents with a “clicking” sound and more than 3 months of sternal instability without associated infection. On CT, a sternal gap can be seen to have well-corticated edges^(2)^, as shown in Figure 9. Transverse fractures and floating bone fragments may also be seen. Up to 50% of patients show a sternal gap within the first six months post-sternotomy, which is not indicative of a pathological process. Therefore, in the absence of symptoms, a diagnosis of sternal non-union should be considered only > 12 months after sternotomy^(1)^.

### Infections

**Figure 10:**
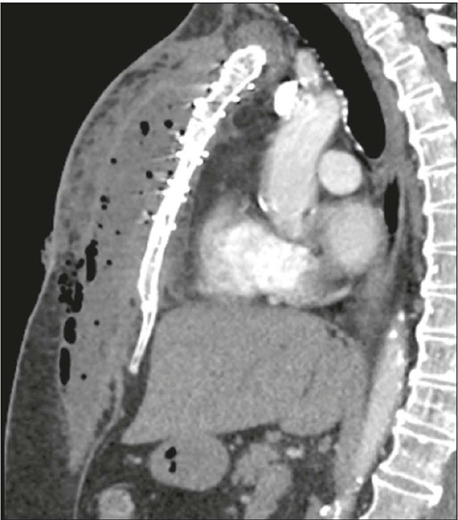
Superficial infection of the surgical wound (sagittal CT image; mediastinal window). Extensive fluid collection with foci of gas in the anterior chest wall (presternal compartment), with densification of the adjacent soft tissues, without extension into the mediastinum.

**Superficial infection** – Superficial wound infection (with an incidence of 0.5–8.0%) involves the skin, subcutaneous tissue, and pectoralis fascia. Symptoms include erythema, fever, purulent drainage, and sternal instability. A CT scan can reveal subcutaneous emphysema, fat stranding, and fluid collections limited to the chest wall (Figure 10). Treatment involves intravenous injection of antibiotics and local care^(7,8,9,10)^.

### Mediastinitis

**Figure 11:**
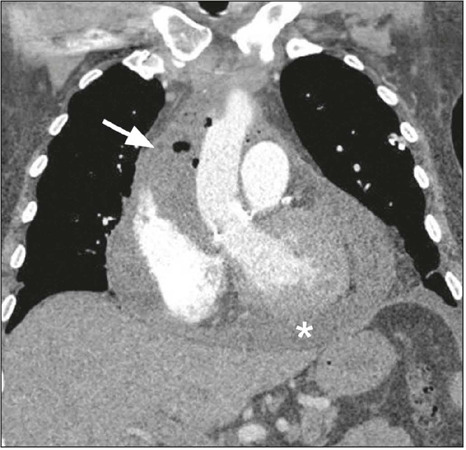
Mediastinitis in a patient with purulent secretion from the wound, increased C-reactive protein and leukocytosis (coronal CT image; mediastinal window). Extensive fluid collection with foci of gas surrounding the ascending aorta (arrow), in contiguity with pericardial effusion (asterisk).

Mediastinitis, which has an incidence of 0.4–5.0%, is a potentially fatal complication, associated with a mortality rate of 12–50%^(8)^. Risk factors include diabetes, obesity, excessive cauterization, prolonged surgery, and internal mammary artery harvest^(1)^. When there is suspicion of early mediastinitis (within the first 2–3 weeks), the clinical manifestation is crucial, given that CT may be inconclusive during this period, with normal postoperative findings resembling pathological ones. Signs and symptoms include fever, leukocytosis, sternal pain/instability, and purulent drainage^(9)^. After 21 days, CT findings such as mediastinal fluid collections (including complex fluid collections), mediastinal fat densification, and pneumomediastinum (Figure 11) become more specific for the diagnosis of mediastinitis^(4)^. Other signs include sternal diastasis, osteomyelitis, mediastinal lymphadenopathy, pleural/pericardial effusion, and pulmonary consolidation^(8^, ^9,10)^. Management involves surgery and long-term antibiotic use^(1)^.

### Retrosternal abscess

**Figure 12:**
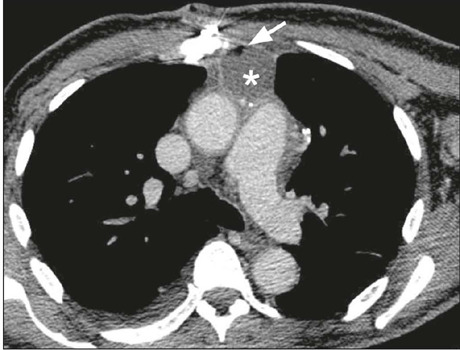
Retrosternal abscess (contrast-enhanced CT image; axial plane; mediastinal window). Organized fluid collection in the prevascular mediastinum (asterisk) containing a focus of gas (arrow) and showing peripheral enhancement, consistent with retrosternal abscess.

Retrosternal abscess is defined as an infected encapsulated fluid collection in the prevascular mediastinum that arises weeks (or months) after sternotomy. It occurs due to hematogenous spread or contiguity. *Staphylococcus* spp. are the most common pathogens. On CT, it appears as a hypodense fluid collection with thick or irregular peripheral enhancement and adjacent fat stranding, with or without foci of gas (Figure 12). Fistulous tracts to the skin, pericardium, or pleura may develop. In case of uncertainty, percutaneous needle aspiration may be performed to differentiate an abscess from a sterile postoperative seroma or hematoma. Definitive treatment often requires surgical exploration and drainage^(1,2)^.

### Osteomyelitis

**Figure 13:**
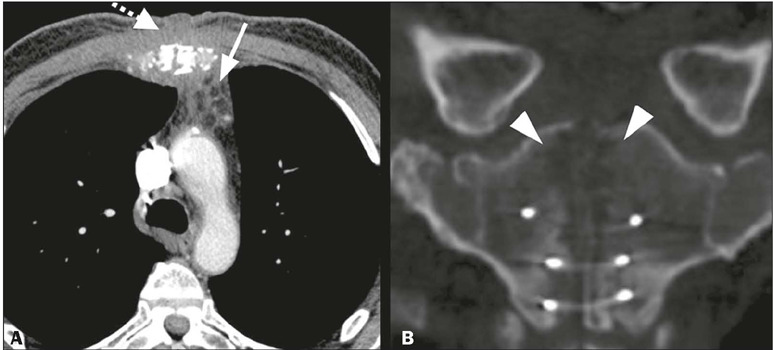
Osteomyelitis. **A:** Axial CT image; mediastinal window, showing densification of the peristernal planes, with fluid accumulations (dashed arrow), and fat stranding in the prevascular mediastinum (arrow). **B:** Coronal CT image; bone window, showing bone demineralization and osteolysis of the right and left fragments of the sternal manubrium (arrowheads), suggestive of osteomyelitis.

**Figure 14:**
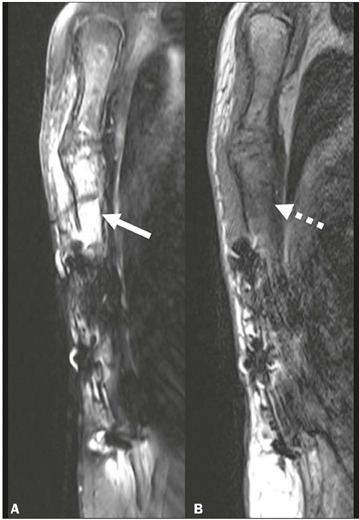
Osteomyelitis depicted by magnetic resonance imaging. **A:** Sagittal T2-weighted SPAIR image showing hyperintense signal within the sternum, predominantly affecting the superior third of the body (arrow), consistent with bone marrow edema. Note also the edema in the surrounding presternal soft tissues. **B:** Sagittal T1-weighted image showing hypointense signal within the sternum, most pronounced in the superior third of the body (dashed arrow), consistent with osteomyelitis. Metallic sternal wires produce susceptibility artifacts, particularly limiting the evaluation of the lower two-thirds of the sternal body.

Post-sternotomy osteomyelitis is a serious complication, with an incidence of 0.5–5.0% and an associated mortality rate of up to 29%, that may arise weeks, months, or years after the surgery. *Staphylococcus* spp. are the main causative agents^(1^, ^2)^. In the recent postoperative period, the diagnosis is challenging because of the usual sternal irregularities caused by surgical manipulation^(2)^. Findings on CT include bone erosion/sclerosis, periosteal reaction, and cortical destruction (Figure 13). Sequestrum, involucrum, abscess, and sinus tract formation may also occur. Osteomyelitis is often associated with sternal wound dehiscence or sternal non-union. Magnetic resonance imaging is the gold standard imaging modality for the diagnosis of osteomyelitis, enabling the detection of pathological bone marrow signal intensity, which includes hypointensity on Tl-weighted images and hyperintensity on T2-weighted images (Figure 14), although its effectiveness can be limited by metal artifacts. Serial CT scans, in conjunction with the clinical and laboratory status of the patient, are often useful for diagnosis. Treatment includes antibiotics, drainage of collections, debridement of necrotic tissue, removal of infected sternal hardware, negative-pressure wound therapy, and chest wall reconstruction^(1,2)^.

## CONCLUSION

**Figure 15:**
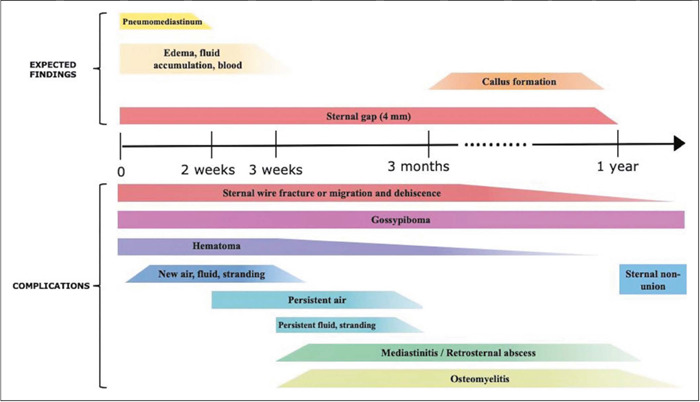
Timeline for expected findings and complications after median sternotomy.

Radiological evaluation of the chest after sternotomy is challenging for radiologists because normal findings due to surgical trauma can resemble complications. The image interpretation should take into consideration the recovery timelines (Figure 15), as well as the clinical and laboratory status of the patient. This approach is essential to achieve an accurate diagnosis and to inform decisions regarding the appropriate management of post-sternotomy complications.
